# Lineup identification in young and older witnesses: does describing the criminal help or hinder?

**DOI:** 10.1186/s41235-022-00399-1

**Published:** 2022-06-17

**Authors:** Juliet S. Holdstock, Polly Dalton, Keith A. May, Stewart Boogert, Laura Mickes

**Affiliations:** 1 Department of Psychology, School of Life Sciences and the Environment, Royal Holloway, University of London, Egham, UK; 2 Department of Psychology, University of Essex, Colchester, UK; 3 Department of Physics, Royal Holloway, University of London, Egham, UK; 4 School of Psychological Science, University of Bristol, Bristol, UK

**Keywords:** Lineup, Ageing, Verbal overshadowing, Eyewitness, Discriminability, Confidence, Response time

## Abstract

The world population is getting older and, as a result, the number of older victims of crime is expected to increase. It is therefore essential to understand how ageing affects eyewitness identification, so procedures can be developed that enable victims of crime of all ages to provide evidence as accurately and reliably as possible. In criminal investigations, witnesses often provide a description of the perpetrator of the crime before later making an identification. While describing the perpetrator prior to making a lineup identification can have a detrimental effect on identification in younger adults, referred to as verbal overshadowing, it is unclear whether older adults are affected in the same way. Our study compared lineup identification of a group of young adults and a group of older adults using the procedure that has consistently revealed verbal overshadowing in young adults. Participants watched a video of a mock crime. Following a 20-min filled delay, they either described the perpetrator or completed a control task. Immediately afterwards, they identified the perpetrator from a lineup, or indicated that the perpetrator was not present, and rated their confidence. We found that describing the perpetrator decreased subsequent correct identification of the perpetrator in both young and older adults. This effect of verbal overshadowing was not explained by a change in discrimination but was consistent with participants adopting a more conservative criterion. Confidence and response time were both found to predict identification accuracy for young and older groups, particularly in the control condition.

## Significance statement

The proportion of the world population aged 65 and above is increasing and is predicted to reach 16% by 2050 (United Nations, 2019). The number of older witnesses to crimes is therefore expected to increase, making it essential that we understand how age affects eyewitness identification so that procedures can be adopted to ensure that older witnesses can provide evidence as accurately and reliably as possible. In criminal investigations, witnesses are interviewed and asked to provide a detailed description of the crime. Perpetrator descriptions provided during these interviews can help the police select a suspect and create identification lineups. It is possible, however, that providing such a description may affect later identification of the suspect. While providing a perpetrator description has been shown to worsen identification accuracy in young individuals under certain circumstances, it is unknown how it affects identification accuracy in older individuals. We found 1) that young and older adults made fewer correct identifications after describing the perpetrator which was explained by an increased reluctance to choose a face from the lineup rather than increased difficulty distinguishing between innocent and guilty suspects—knowledge of particular relevance to policymakers who determine the procedures used for acquiring evidence; 2) that the confidence and the speed with which both young and older witnesses made their identification predicted their identification accuracy, particularly when they were not required to provide a description—knowledge which is likely to be of primary importance to the judge and jurors in the courtroom.

## Introduction

The world population is ageing (United Nations, 2019) and as a result the number of older victims of crime is expected to increase. It is therefore essential to understand eyewitness identification throughout the lifespan so procedures can be developed that enable victims of crime of all ages to provide evidence as accurately and reliably as possible. This will result in better identifications of perpetrators of crime while reducing the number of misidentifications of innocent people as perpetrators.

In criminal investigations, witnesses are interviewed about the crime to produce a witness statement. As part of this interview the witness is asked to provide a full detailed physical description of any people, they mention during their recount of the crime starting with the suspect (Metropolitan Police, 2018). A detailed description of the perpetrator can be used by the police to help identify a suspect and create an identification lineup. It is not unusual for a witness to provide a detailed description of the perpetrator and then shortly afterwards to be asked to view photographs held by the police that fit the description or that are of suspects who have committed other similar crimes in that area (see Memon & Bartlett, 2002). The question has arisen as to how providing a detailed description of the perpetrator’s face affects the ability of the witness to later identify them. This study investigated whether describing the perpetrator prior to making a lineup identification affected older adults in the same way as younger adults.

In an influential study, young adults watched a video of a staged bank robbery and were subsequently asked to try to identify the perpetrator from a lineup comprising the perpetrator’s face and 7 filler faces whose descriptions matched that of the perpetrator (Schooler & Engstler-Schooler, 1990). When the participants provided a detailed description of the perpetrator prior to the administration of an identification procedure, they were less likely to select the perpetrator from the lineup compared to participants who completed an unrelated verbal (control) task prior to the administration of an identification procedure. This decrement in correct identification was termed “verbal overshadowing” (Schooler & Engstler-Schooler, 1990).

While verbal overshadowing was found in some subsequent studies, it has not been consistently found, and a meta-analysis revealed effect sizes that were smaller than those of the original study (Meissner & Brigham, 2001). As a result, a large registered replication study (Alogna et al., 2014), involving multiple laboratories, was conducted on two experiments reported in Schooler and Engstler-Schooler (1990). In one experiment, participants described the perpetrator or completed a control task 20 min after watching the video of the staged bank robbery and immediately before the administration of the lineup procedure. Similar to the results of the original study, the replication effort found that the correct identification rate was significantly lower when the perpetrator was described relative to the “no description” control condition. In the other experiment, participants described the perpetrator or completed the control task immediately after viewing the video and 20 min before the administration of the lineup procedure. The replication effort found a much smaller effect on correct identification rates in this configuration than were found in the original study.

The effect of verbal overshadowing on the correct identification rate on a lineup task does therefore appear to be consistently found in young adults when an interval of 20 min intervenes between the video of the mock crime and description of the perpetrator. However, it is difficult to interpret this finding because changes in correct identification rates could be due to either an effect on recognition memory per se, i.e. difficulty distinguishing between innocent and guilty suspects (discriminability), or a reduction in willingness to make an identification (more conservative response bias) (Clare & Lewandowsky, 2004; Gronlund et al., 2014; Wixted & Mickes, 2014; Wixted et al., 2014). Correct identification rates should be jointly considered with false identification rates before claims about a reduction in memory are made (Mickes, 2016; Mickes & Wixted, 2015; Rotello et al., 2015).

To distinguish between these two interpretations, new methods were developed for analysing eyewitness identification data that provided separate measures of discriminability and response bias (Wixted & Mickes, 2014). Using these new analyses, Wilson et al. (2018) replicated the same two experiments reported in Schooler and Engstler-Schooler (1990) that were replicated in the large study of Alogna et al. (2014). However, one slight, but critical, adaptation was made. Half of the participants in each experiment were asked to try to select the perpetrator from a lineup in which the perpetrator was present (target-present lineup), as in the previous studies, but the other half were asked to try to select the perpetrator from a lineup in which the perpetrator was not present (target-absent lineup). This enabled the calculation of false identification rate as well as correct identification rate which enabled discriminability to be ascertained separately from response bias.

Verbally describing the perpetrator did reduce discriminability, but only when a filled 20-min delay intervened between the video of the mock crime and the description. Providing a verbal description immediately after the video did not affect discriminability (Wilson et al., 2018). These findings therefore suggested that recognition memory itself, i.e. the ability to distinguish between guilty and innocent suspects, was reduced by describing the perpetrator but only when the verbal description was provided 20 min after viewing the mock crime (and right before the lineup test was taken).

This finding is consistent with the diagnostic feature detection theory (Wixted & Mickes, 2014) according to which discriminability is better when individuals rely on more diagnostic features than when they rely on less diagnostic features that are shared by all of the lineup members. This theory was used to explain the findings reported in Wilson et al. (2018). The verbal descriptions given after a short delay, which did not result in verbal overshadowing, contained more diagnostic features that were shown to be useful in selecting the perpetrator. The verbal descriptions provided after a longer delay, which resulted in verbal overshadowing, contained fewer diagnostic features and more generic features which were shared by all lineup members and so reliance on this verbal description to some extent during identification would not help in selecting the perpetrator. It has been argued that providing a verbal description could lead to a shift from non-verbal to verbal processing and reliance on verbal processing during identification (Brown & Lloyd-Jones, 2002; Brown & Lloyd‐Jones, 2003; Melcher & Schooler, 2004) or could result in interference with and re-encoding of the non-verbal representation (Meissner & Brigham, 2001; Schooler & Engstler-Schooler, 1990). According to the diagnostic feature detection theory, however, the verbal description only has a detrimental effect on identification and results in verbal overshadowing if it contains few diagnostic features and many features shared by lineup members, as found by Wilson et al. (2018). This was supported by a recent computational model of the recoding interference hypothesis that was unable to discriminate between old faces (on which the model had been trained) and new (untrained) faces when the verbal description given to the model contained information consistent with both faces (Hatano et al., 2015).

### Older adults

All of the participants in the previously mentioned studies were young adults. Much less research on verbal overshadowing has been conducted on older adult participants. Studies involving this age group have found no significant effect of verbal overshadowing on correct identification in both young and older adults (Kinlen et al., 2007; Memon & Bartlett, 2002). Kinlen et al. (2007) did, however, report that their older group performed significantly better than their younger group in the verbalisation condition but not in the control condition. In both of these studies the findings are difficult to interpret because analytical methods to distinguish between discriminability and response bias were not used and there were very small sample sizes which has greatly limited their power. These data are therefore only suggestive and should be treated with caution, but nevertheless, the findings of Kinlen et al. (2007) raise the interesting possibility that verbally describing the perpetrator may have less of a detrimental effect on older adults than young adults potentially eliminating the verbal overshadowing effect and perhaps even facilitating memory.

Such an effect might be expected from other findings. Face recognition has been shown to depend on configural processing, i.e. processing the spatial distances between face features (Maurer et al., 2002; Tanaka & Farah, 1993). However, in older adults impairments in configural processing of faces have been reported (Chang et al., 2019; Daniel & Bentin, 2012; Gao et al., 2009; Obermeyer et al., 2012; Slessor et al., 2013). As disruption of configural processing has been argued to reduce verbal overshadowing (Fallshore & Schooler, 1995), verbal overshadowing might not be expected in older adults.

An absence or reduction in verbal overshadowing in older adults may also be expected based on findings from another line of research. Some research suggests that older adults may have better vocabulary and general knowledge than younger adults (Foos & Sarno, 1998; Hartshorne & Germine, 2015; Kausler, 1991; Long & Shaw, 2000). This may result in older adults having a better ability to describe the diagnostic features of the faces than younger adults which could lead to a reduction or absence of verbal overshadowing.

Our study tested the prediction, arising from the previous literature, that while describing the perpetrator results in verbal overshadowing in young adults, it may not result in verbal overshadowing in older adults. We used the procedure that resulted in a verbal overshadowing effect in young adults in Wilson et al. (2018). This enabled us to determine whether or not older adults showed a verbal overshadowing effect under the conditions in which verbal overshadowing has been consistently shown in young adults. To overcome the limitations of previous studies, large samples of young and older adults were compared. Furthermore, we analysed the data using measures that distinguished between discriminability and response bias in lineup data (Mickes et al., 2012; Wixted & Mickes, 2014).

While these measures have not yet been used to study the effect of verbal overshadowing on lineup identification in older adults, they have been used to investigate lineup identification in older adults under standard conditions, when no description of the perpetrator was required. Discriminability was poorer in older (60–95 years of age) than younger (aged 18–30 years) adults (Colloff et al., 2017). This finding is in line with a body of literature showing that episodic memory is reduced in older adults (e.g. Hedden & Gabrieli, 2004; Nyberg & Tulving, 1996; Nyberg et al., 2003; Rönnlund et al., 2005). Although consistent with a deficit in episodic memory, it is also possible that the poorer discriminability of older adults (Colloff et al., 2017) resulted from, or was in part affected by, the own-age effect. Individuals are better at recognising faces from their own age group than other age groups (Rhodes & Anastasi, 2012). Young adults have been reported to recognise photographs of young adults better than photographs of older adults and older adults have been reported to recognise photographs of older adults better than photographs of young adults (e.g. Anastasi & Rhodes, 2006; Rhodes & Anastasi, 2012). It is therefore possible that by using a young adult as the perpetrator in the mock crime video (Colloff et al., 2017), the older participants were unwittingly disadvantaged. To circumvent this concern, we used a middle-aged actor and lineup members so that neither young nor older individuals benefited from an own-age effect. Both young and older adults have been reported to recognise photographs of middle-aged adults as well as photographs of adults of their own-age (Anastasi & Rhodes, 2006; Cronin et al., 2020; Randall et al., 2012), but see Wolff et al. (2012). Although not the primary focus of this study, the use of a middle-aged perpetrator enabled us to examine whether discriminability of older adults remained poor when own-age effects were controlled in this way. If the previously reported poorer discriminability of older adults (Colloff et al., 2017) was due to poorer episodic memory, we predicted that in our control condition, which like Colloff et al. (2017) did not require description of the perpetrator, discriminability would be poorer in our older adult group than our young adult group. In contrast, if it was due entirely to an own-age effect, no differences between the discriminability of the older adult group and younger adult group would be predicted in our control condition.

### Predictors of suspect identification accuracy

*Confidence*. Much of the focus of verbal overshadowing has been on reduced correct identification rates or reduced discriminability. While knowledge about witness discriminability is of importance to policymakers, who determine the procedures used for acquiring evidence; for the judge and jurors in the courtroom, information that indicates how accurate a witness’s response is likely to be is of primary importance (Mickes, 2015, 2016). The confidence of young adult witnesses when they make an initial identification has been shown to be informative about the accuracy of their choice (Brewer & Wells, 2006; Grabman et al., 2019; Juslin et al., 1996; Seale-Carlisle et al., 2019a, 2019b; Semmler et al., 2018; Wilson et al., 2018). This has also been reported with older adults when identification is made after a short delay, even when their overall discriminability is lower than younger adults (Colloff et al., 2017).

Our study aimed to determine whether this association between confidence and accuracy replicated in older adults with a slightly longer delay and how it was affected by providing a verbal description of the perpetrator. In young adults, describing the perpetrator made no significant difference to the relationship between confidence and accuracy of identification (Wilson et al., 2018). Our study also allowed for replication of these findings in a different sample of younger adults.

*Response time.* It has also been shown that, for young adult witnesses, the speed with which initial lineup identification decisions are made is informative about the accuracy of their responses with faster responses being more accurate (e.g. Brewer et al., 2006; Dodson & Dobolyi, 2016; Dunning & Perretta, 2002; Sauerland & Sporer, 2009; Seale-Carlisle et al., 2019a, 2019b; Smith et al., 2001; Sporer, 1992, 1993; Weber et al., 2004). Furthermore, response time and confidence together predicted suspect identification accuracy better than each alone (Seale-Carlisle et al., 2019a, 2019b). For a particular level of confidence suspect identification accuracy was higher when the response was made quickly than when it was made slowly (Seale-Carlisle et al., 2019a, 2019b). We, therefore, also measured the time taken by participants to make their identification decision on the lineup task to assess whether response time predicted the accuracy of identification in older as well as younger adults.

To summarise, our study aimed to answer a number of questions concerning lineup identification performance of older vs. younger adults. In terms of discriminability,

1. When no verbal description was required (i.e. the control condition), would discriminability of older adults be lower than that of young adults when own-age effects were controlled by using a middle-aged perpetrator and lineup members?
2. Would providing verbal descriptions have different effects on the discriminability of older adults and young adults?

In terms of predictors of suspect identification accuracy,

1. Would high confidence suspect identifications be higher in accuracy than lower confidence suspect identifications for both older adults and young adults?
2. Would the confidence–accuracy relationship be unaffected by providing a verbal description of the perpetrator for both older adults and young adults?
3. Would identifications made quickly be more accurate than those made more slowly for both older adults and young adults?
4. Would the response time–accuracy relationship be unaffected by providing a verbal description of the perpetrator for both older adults and young adults?

## Methods

We submitted this article as a Registered Report. The hypotheses, methods, and analysis plans were subjected to peer review and accepted at Stage 1. We then created stimuli, collected data, conducted analyses, and wrote the rest of the manuscript to complete Stage 2. The entire manuscript was then peer-reviewed. The introduction, method, and analysis strategy sections have not changed after Stage 1 except for verb tenses that went from future to past. Any other changes are specified in the text.

### Participants

A sample of 1000 healthy young participants aged between 18 and 30 years and a sample of 1000 healthy older participants aged 60 years and older were recruited. Participants were excluded if they self-reported having a diagnosis of a neurological disorder that affects their memory, e.g. mild cognitive impairment, Alzheimer’s disease (Koen & Yonelinas, 2014). Half of the participants (*n* = 500) from each group were randomly allocated to the experimental condition where they described the perpetrator, and half (*n* = 500) were randomly allocated to the control condition where they wrote the names of capital cities and their corresponding countries.

A sample size of 500 participants per condition is comparable to previous forensically relevant studies that have found verbal overshadowing in young adults using the same procedure as our study (Wilson et al., 2018) and have demonstrated the confidence–accuracy relationship (Mickes, 2015). A power analysis conducted using pyWitness [https://lmickes.github.io/pyWitness/] and data from Experiment 2 of Wilson et al. (2018) showed that our study had sufficient power with a sample of this size. The data were fitted to an equal variance signal detection model which was used to simulate synthetic data with a variable number of participants. For each synthetic dataset, a complete ROC analysis was conducted, including the calculation of pAUC values. The standard error of the pAUCs was estimated using the bootstrap method with 2,000 replicates. The pAUC between control and experimental conditions were then tested by calculating

$$Z \, = \, \left( {{\text{pAUC\_1 - pAUC\_2}}} \right){\text{ / sd}}\left( {{\text{pAUC\_1 - pAUC\_2}}} \right)$$

and using this Z to compute two-sided *p* values. For *n* = 500, *Z* = 1.893, *p* = 0.00056.

Half of the participants in each condition were randomly allocated a target-present lineup and half were randomly allocated a target-absent lineup. Data collection ceased once we had data from 500 participants per condition. The study had ethical approval from Royal Holloway, University of London. All participants provided informed consent prior to taking part in the study.

All participants were recruited through the Prolific online participant recruitment platform.

### Materials

We created a 25 s video of a non-violent, mock crime, showing a white middle-aged male perpetrator stealing a handbag from a parked car. The chronological age of the perpetrator was 49 years. Eighteen participants aged between 20 and 62 years old estimated his age and gave a modal rating of 40–49 years old, consistent with his chronological age. His age therefore fell outside of the age range of both our young and older participant groups, thus counteracting the own-age effect. He did not have any distinctive distinguishing features.

For the lineup tasks we used a photograph of the perpetrator and a pool of 36 filler faces selected from The Chicago Face Database (Ma et al., 2015), Face Research Lab London Set (DeBruine & Jones, 2017), Utrecht ECVP database (2008), and the face database from the Park Aging Mind Laboratory (Minear & Park, 2004) that were matched in perceived age to the rated age of the perpetrator (40–49 years). Following the recommendations of Wells et al. (2020), the filler faces were selected to match a description of the perpetrator given by participants (*N* = 18) who watched the video and answered questions about the perpetrator’s appearance. To ensure that the photograph of the suspect did not stand out at all (Wells et al., 2020), all the photographs were edited to show just the face and hair within an oval window and with a grey background.

Two lineups were constructed, each of which comprised six simultaneously presented face photographs arranged as two rows of three faces. A “Not Present” button was present to the right of the faces. In the target-present lineup the faces comprised the perpetrator and five filler faces randomly selected from the filler face pool. In the target-absent lineup six filler faces were presented which were randomly selected from the filler pool. The position in which the target (perpetrator) face appeared in the target-present lineup was randomly determined for each participant.

To prevent rehearsal of the video during the 20-min retention interval participants completed a distractor task. The distractor task was solving anagram puzzles (Colloff et al., 2017; Morgan et al., 2019).

### Design

Each participant watched the same video and then completed one of the two conditions. Half of the participants in each age group were randomly allocated to the experimental condition and half were randomly allocated to the control condition. For each of these conditions half of the participants were randomly allocated a target-present lineup and half were allocated a target-absent lineup.

### Procedure

The Gorilla Experiment Builder (www.gorilla.sc) was used to create and host our experiment (Anwyl-Irvine et al., 2020). Participants were restricted, by the Gorilla software, to accessing the experiment on a PC or laptop and were not able to access it using a mobile phone or tablet.

Participants viewed the video of the mock crime. They were instructed to attend well so they could answer questions about it later. The video was followed by a 20-min delay during which participants completed the distractor task (solving anagram puzzles). In the experimental condition, participants were given five minutes to type a detailed description of the perpetrator’s appearance. Following Wilson et al. (2018), participants in this condition were given the following instructions from Alogna et al. (2014): “Please describe the appearance of the bank robber in as much detail as possible. It is important that you attempt to describe all of his different facial features. Please write down everything that you can think of regarding the bank robber’s appearance. It is important that you try to describe him for the full 5 min” (pp. 559–560) with “bank robber” replaced with “perpetrator of the crime”. In the control condition participants instead typed the names of as many capital cities and their countries as they could within five minutes. Immediately following this, participants were instructed that they would now be shown a lineup in which the person from the video may or may not be present. They were then presented with either a target-present or target-absent lineup showing six faces and a “not present” option. Participants selected the face of the perpetrator from the lineup or selected “not present” if the perpetrator was not present. The time from the appearance of the lineup on the screen until the participant made a button press to indicate their response was recorded (response time). Participants also indicated their confidence on a 7-point scale (1 = guessing; 7 = certain). Finally, as an attention check, participants answered the multiple-choice question, “What crime was committed in the video?”. Demographic information (age, ethnicity, nationality, gender, and years in full-time education) was also collected.

## Analysis strategy

Participants who incorrectly answered the attention check question, reported a technical problem, or reported that they had not followed the lineup test instructions were excluded from all analyses. They were replaced to achieve the desired sample size (*N* = 2000). Alpha levels were set to 0.05 and Bonferroni corrections were used for multiple comparisons. The data are available at https://doi.org/10.17605/OSF.IO/7EA23. The analysis code for pyWitness is also available for reproducibility and extension. All analyses, plots, and model fits were conducted using pyWitness (https://lmickes.github.io/pyWitness/; Mickes et al., 2022).

### Correct and false ID rates

Correct ID rates were computed from the target-present lineups. Correct ID rates were calculated by dividing the number of times the perpetrator was successfully identified by the total number of target-present lineups. False ID rates were calculated from the target-absent lineup. As we did not have a designated innocent suspect, the false ID rate was estimated, which is standard practice (e.g. Palmer et al., 2013). The estimated false ID rates were computed by dividing the number of faces incorrectly identified in the target-absent lineup by the total number of target-absent lineups and then dividing by the number of lineup members (6).

### Discriminability

*Empirical discriminability.* To measure discriminability, we conducted confidence-based receiver operating characteristic (ROC) analysis (Gronlund et al., 2014; Mickes, 2015; Mickes et al., 2012). This commonly used approach plots the correct ID rate and false ID rate for each level of confidence resulting in ROC curves per condition or group. To measure differences, partial area under the curve (pAUC) was computed for each group and condition (Gronlund et al., 2014). In order to measure pAUC, a false ID cut-off has to be specified. We took as the cut-off the rightmost point on the ROC from the condition that yielded more conservative responding overall.

To test if we replicated the effect that older adults have lower discriminability than younger adults when verbal description of the perpetrator is not required (Colloff et al., 2017), we compared pAUC of the older group with the pAUC of the younger group in the control condition. To test if we replicated the verbal overshadowing effect for young adults reported in Wilson et al. (2018), we compared pAUC of the experimental group with the pAUC of the control group for the young participants. To test if the older adults showed a verbal overshadowing effect, we compared pAUC of the experimental group with the pAUC of the control group for the older participants. All pAUC comparisons were made using *Z*. *Z* is equal to the pAUC difference divided by the standard error of the pAUC differences (Robin et al., 2011). The standard errors were estimated using bootstrapping. (The number of bootstraps were set to 10,000.) Given the number of comparisons, Bonferroni corrections were used.

### Predictors of suspect identification accuracy

*Confidence.* To test whether confidence predicted suspect ID accuracy we conducted confidence–accuracy characteristic (CAC) analysis (Mickes, 2015). In CAC analysis identification accuracy is computed separately for every level of confidence. For a six-person lineup with no designated innocent suspect, as in the current study, CAC is given by

$${\text{CAC = }}\frac{{{\text{CID}}_{{{\text{conf}}}} }}{{{\text{CID}}_{{{\text{conf}}}} {\text{ + FID}}_{{{\text{conf}}}} {/6}}}$$

where CID_conf_ is the number of suspect IDs made with a particular level of confidence from the target-present lineup and FID_conf_ is the number of filler IDs made with the same level of confidence from the target-absent lineup, which is divided by the number of people in the lineup (6 in this case).

A bootstrap procedure was used to estimate the standard errors associated with suspect ID accuracy for each level of confidence for each condition. Observed data on target-present and target-absent lineups were randomly sampled with replacement to obtain a bootstrap sample for each trial. This was repeated for 10,000 bootstrap trials and we used 68% bootstrap confidence intervals to estimate ± 1 standard error. This was performed separately for each condition. Non-overlapping error bars were interpreted as a reliable difference (Seale-Carlisle & Mickes, 2016).

Separate CAC analyses were conducted for the young and older groups and for the description and control conditions. These analyses tested the prediction that suspect identification accuracy increased with confidence in both the older and younger participants. They also tested whether performing the verbal description task affected this relationship and whether it affected it differently in the two age groups.

*Response time.* The relationship between response time and identification accuracy was investigated using a procedure similar to the CAC analysis. The response-time accuracy characteristic (RAC) analysis was developed by Seale-Carlisle et al. (2019), Seale-Carlisle et al. (2019)) to determine whether response time predicts suspect identification accuracy. Following the procedure of Seale-Carlisle et al. (2019a, 2019b), identification responses were binned according to how fast they were made (< 5 s, 6–15 s, 16–30 s, > 30 s) and suspect identification accuracy (i.e. proportion correct) was calculated separately for each response speed bin. This was done separately for each age group and for each condition (description, control).

For a six-person lineup, RAC is given by

$${\text{RAC = }}\frac{{{\text{CID}}_{{{\text{RT}}}} }}{{{\text{CID}}_{{{\text{RT}}}} {\text{ + FID}}_{{{\text{RT}}}} {/6}}}$$

where RT is response time, CID_RT_ is number of suspect IDs made with a particular response time from the target-present lineup (e.g. the number of suspect IDs made with RT < 5 s) and FID_RT_ is the number of filler IDs made with a particular response time from the target-absent lineup, which is divided by the number of people in the lineup (6 in the current experiment). The bootstrap procedure described for the confidence–accuracy analysis was used to estimate the standard errors associated with suspect ID accuracy for each response time bin for each condition. Non-overlapping error bars were interpreted as a reliable difference.

These analyses tested our prediction that response time decreased as accuracy increased in both the older and younger participants (i.e. faster responses were more accurate than slower responses). The analyses also tested whether performing the verbal description task affected this relationship and whether it affected it differently in the two age groups.

### Analyses that were planned but were not included

The analysis of underlying discriminability described below was planned to investigate whether any effects of verbal overshadowing on discriminability identified in the empirical ROC analyses differed in magnitude between the two age groups. However, as will be seen in the results section below, no significant effect of verbal overshadowing on discriminability was found in either age group, so this analysis was no longer relevant and was not conducted.

*Underlying discriminability*. We fitted the independent observations signal-detection model to the data (Wixted & Mickes, 2014; Wixted et al., 2018). Fitting a signal detection model to the data yielded parameter estimates (target and lure means and target and lure sigmas), which were used to calculate d′ values (we had planned to use d_a_ values if an unequal variance model had provided a better fit) across a range of criteria. These model-generated d′ (or d_a_) estimates would have been used as input to conduct an analysis of variance (Mickes et al., 2021). With the predicted d′ (or d_a_) values from the model fits, we would have conducted a 2 (older vs. younger) × 2 (experimental vs. control) analysis of variance. This analysis would have allowed us to test for an interaction between experimental vs. control conditions on the different age groups. This analysis, however, was unnecessary given the results described later. Another reason for conducting and comparing the fits was to see whether these results agreed with the pAUC results (Wixted & Mickes, 2018), which they did. We fitted the model and used the model fits for the curves in the ROC plots, but as this paper is not a modelling paper, we do not address this further.

## Results

The demographic data for the young and older groups are shown in Table 1. All participants were resident in the UK at the time of participation. Six young and seven older participants were excluded and replaced to achieve 1000 participants per age group (250 per condition and lineup type). Participants were excluded because they failed the attention check (2 young participants), reported an age outside the specified age range (2 young and 3 older participants), did not follow the instructions (2 young and 3 older participants) or reported a technical problem (1 older participant).

**Table 1:** Demographic data for young and older groups

	Group	
	Young Adults	Older Adults
Gender (percentage)		
Female	57.4	58.4
Male	40.1	41.5
Transgender	0.4	0
Non-binary	2	0.1
Other	0.1	0
Age (years)		
Mean (standard deviation)	23.86 (3.7)	65.15 (4.89)
Range	18–30	60–89
Ethnicity (percentage)		
White	80.5	97.2
Black	4	0.6
Latino/Latina	0.3	0.1
South Asian	6.6	0.7
East Asian	2.4	0.5
Middle Eastern	0.8	0.1
Arab	0.7	0
Other	4.7	0.8
Education (years)		
Mean (standard deviation)	15.41 (3.46)	14.5 (3.21)

### Correct and false ID rates

Table 2 shows for both age groups the frequencies of correct identifications, filler identifications and no identifications at each level of confidence for the control and experimental conditions. The correct ID rates for the control and experimental conditions were 0.604 and 0.44, respectively, for the young group, and 0.368 and 0.20, respectively, for the older group. The estimated innocent suspect false ID rates for the control and experimental conditions were 0.056 and 0.031, respectively, for the young group, and 0.061 and 0.036, respectively, for the older group.

**Table 2:** Frequencies of correct identifications (CID), filler identifications (FID), and no identifications (No ID) for each age group at each level of confidence for the control and experimental conditions

	1	2	3	4	5	6	7
Young							
Control							
*Target present*							
CID	0	2	11	15	43	37	43
FID	2	1	2	5	10	2	1
No ID	0	1	8	12	31	16	8
*Target absent*							
FID	2	3	21	18	35	3	2
No ID	3	5	19	24	60	34	21
Experimental							
*Target present*							
CID	0	2	7	21	39	27	14
FID	0	2	5	11	4	2	0
No ID	2	5	10	23	40	20	16
*Target absent*							
FID	2	6	7	12	17	3	0
No ID	3	7	24	22	59	55	33
Older							
Control							
*Target present*							
CID	0	4	2	19	35	20	12
FID	2	3	3	14	18	6	0
No ID	3	1	9	22	40	29	8
*Target absent*							
FID	1	8	10	23	37	10	2
No ID	3	5	9	27	45	47	23
Experimental							
*Target present*							
CID	0	1	5	8	18	11	7
FID	0	4	9	3	8	3	1
No ID	3	10	8	35	48	41	27
*Target absent*							
FID	1	4	5	16	23	4	1
No ID	1	5	12	27	58	50	43

Unplanned analyses were conducted to explore further how correct identifications from the target-present lineups and false identifications from the target-absent lineups differed between the control and experimental conditions. These showed that both age groups made significantly fewer correct and false IDs in the experimental condition. The number of correct identifications made for the target-present lineups in the control and experimental conditions was compared separately for the young and older groups using chi squared. Both young and older participants made significantly fewer correct identifications in the experimental condition than the control condition (χ^2^ = 13.4741, *p* = 0.000242 for the young group; χ^2^ = 17.3499, *p* = 0.000031 for the older group).

The total number of false alarms from the target-absent lineups in the control and experimental condition were also compared separately for the young and older participants. Both age groups made significantly fewer false alarms in the experimental condition than the control condition (χ^2^ = 14.1604, *p* = 0.000168 for the young group; χ^2^ = 13.2977, *p* = 0.000266 for the older group).

### Discriminability

#### Empirical discriminability

**Fig. 1:**
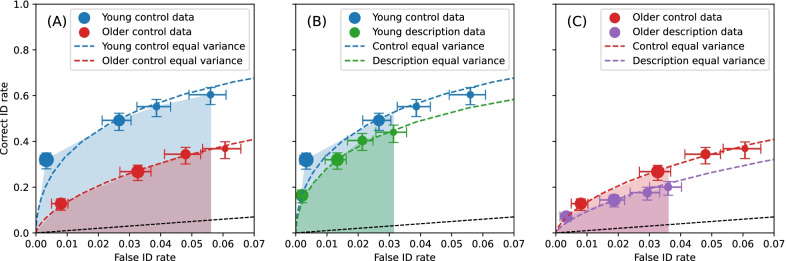
ROC curves for the **A** young and older groups in the control condition, **B** control and experimental conditions for the young group, and **C** control and experimental conditions for the older group. The data were binned into 4 levels of confidence: high confidence (ratings of 6 and 7), medium–high confidence (rating of 5), medium–low confidence (rating of 4), low confidence (ratings of 3, 2 and 1). The size of each data point represents the relative frequency of the responses at each level of confidence. The region used for calculating pAUC for each condition is shown by the shaded regions. The dashed curves represent signal detection model fits (equal variance independent observation model). The error bars are 68% bootstrap confidence intervals, which are the bootstrap equivalent of ± 1 standard error. The black dashed line indicates chance performance

As there were too few responses for the highest and lowest confidence bins to perform bootstrapping, responses were binned in the following manner: low confidence (ratings of 1, 2, and 3), medium–low confidence (rating of 4), medium–high confidence (rating of 5), and high confidence (ratings of 6 and 7). These four confidence bins were used for the ROC analysis and for the CAC analysis which is described later. Figure 1 plots the ROC curves for the comparisons of: A) the young and older groups for the control condition; B) control and experimental conditions for the young group; and C) control and experimental conditions for the older group, respectively. The size of each data point reflects the relative frequency of responses per point (Seale-Carlisle et al., 2019a, 2019b). A larger pAUC indicates better discriminability. The pAUC was significantly smaller for the older group than the young group in the control condition (pAUC young 0.0334 (± 0.0081), pAUC older 0.0138 (± 0.0018), *Z* = 2.3552, *p* = 0.0185). The comparisons of pAUC for control and experimental conditions revealed no significant differences for the young group (pAUC control condition 0.016 (± 0.0057), pAUC experimental condition 0.0114 (± 0.002), *Z* = 0.7589, *p* = 0.4479) or the older group (pAUC control condition 0.0068 (± 0.0012), pAUC experimental condition 0.0053 (± 0.0012), *Z* = 0.8901, *p* = 0.3734).

### Predictors of suspect identification accuracy

#### Confidence

**Fig. 2:**
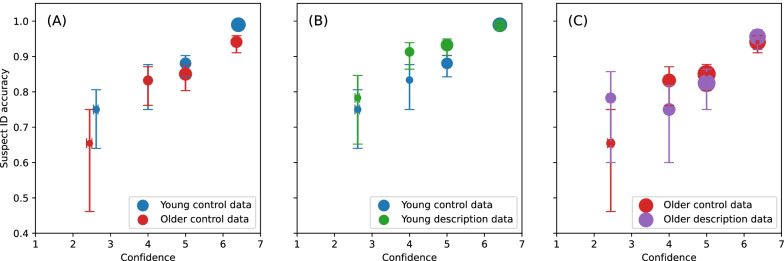
CAC curves showing data for 4 levels of confidence: high confidence (rating of 6 and 7), medium–high confidence (rating of 5), medium–low confidence (rating of 4), and low confidence (rating of 3, 2, and 1) for the **A** young and older group for the control condition, **B** control and experimental conditions for the young group, and **C** control and experimental conditions for the older group. The size of each data point represents the relative frequency of the response. The error bars plot 68% bootstrap confidence intervals, which are the bootstrap equivalent of ± 1 standard error

CAC is a graphical analysis and non-overlapping error bars are taken to indicate reliable differences. Figure 2A shows the CAC curves for the young and older groups in the control condition and shows clearly that the relationship between suspect ID accuracy and confidence was comparable for older and young participants. The bin centres and horizontal uncertainties reflect the averaging from the binning, as seen in the offset points at the lowest confidence for the young and older groups.

CAC curves for the control and experimental conditions are shown for the young group in Fig. 2B. Suspect ID accuracy decreased with decreasing confidence. Accuracy was reliably higher for the high confidence bin (confidence ratings of 6 and 7) than for the three lower confidence bins in both the control and experimental conditions. Suspect ID accuracy did not differ reliably between the three lower confidence bins in the control condition. Accuracy was reliably higher for the two medium confidence bins than for the lowest confidence bin for the experimental condition.

Figure 2C shows the CAC curves for the control and experimental conditions for the older group. As with the young group, suspect ID accuracy decreased as confidence decreased. Suspect ID accuracy in the control condition was reliably higher for the high confidence bin (confidence ratings of 6 and 7) than for the three lower confidence bins and was reliably higher for the two medium confidence bins than for the lowest confidence bin. In the experimental condition, suspect ID accuracy was reliably higher for the high confidence bin than for the three lower confidence bins.

#### Response time

**Fig. 3:**
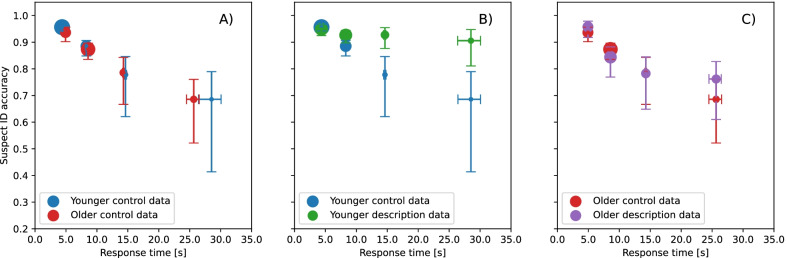
RAC curves for response time bins: < 6 s, 6–12 s, 12–18 s, > 18 s for the **A** young and older group for the control condition, **B** control and experimental conditions for the young group, **C** control and experimental conditions for the older group. The error bars plot 68% bootstrap confidence intervals, which are the bootstrap equivalent of ± 1 standard error

When our data were binned according to precedence by using the response time bins used by Seale-Carlisle et al. (2019a, 2019b), there were insufficient trials for calculating bootstrap error bars in the > 30 s bin for both age groups and in the < 5 s bin in the older group. The data were therefore re-binned to ensure sufficient trials in each bin for each participant group. These new response time bins were < 6 s, 6–12 s, 12–18 s, > 18 s. As with CAC plots, RAC is a graphical analysis and non-overlapping error bars are taken to indicate a reliable difference. Figure 3A plots the RAC curves for the young and older groups for the control condition and shows that suspect ID accuracy decreased with increasing response time in the same way in the two age groups.

Figure 3B plots the RAC curves for the control and experimental conditions for the young group. In the control condition, suspect ID accuracy was reliably higher for the fastest response times of < 6 s than for response times of 6–12 s, 12–18 s and > 18 s. Suspect ID accuracy did not differ reliably between response times of 6–12 s, 12–18 s and > 18 s. In the experimental condition, suspect ID accuracy did not differ reliably between any of the response time bins.

The RAC curves for the control and experimental conditions for the older group are shown in Fig. 3C. For the control condition, suspect ID accuracy was reliably higher for fastest response times of < 6 s and of 6–12 s than for slower response times of > 18 s. For the experimental condition, suspect ID accuracy was reliably higher for responses times of < 6 s than for response times of 6–12 s, 12–18 s and > 18 s. Suspect ID accuracy did not differ reliably between response times of 6–12 s, 12–18 s and > 18 s.

## Discussion

Our study explored eyewitness identification performance of older and young participants when they provided a verbal description of the perpetrator and when they did not. To minimise the own-age effect, we created and used stimuli that would not disadvantage participants in either age group. We found that older participants were poorer at discriminating between the perpetrator and innocent suspects than young participants; that describing the perpetrator decreased correct identification and false identifications in both age groups but did not significantly decrease discriminability; and that higher confidence and faster response time was associated with higher identification accuracy for both groups, with this association holding most consistently in the control condition. We discuss each of these findings in more detail below.

### Discriminability

#### Did discriminability differ between older and young adults when own-age effects were controlled?

Consistent with previous work (Colloff et al., 2017), the older adults in our study were poorer than young adults at discriminating the perpetrator from innocent faces on the lineup task. Considering data from the control condition, which was the condition from our study most comparable to that of Colloff et al. (2017), we found that pAUC was significantly smaller for the older than the young participants. In the Colloff et al. (2017) study, the young age of the perpetrator meant that an own-age effect could not be ruled out as a possible explanation for the difference in 11 between the young and older participants. This explanation is unlikely in our study as we used a middle-aged perpetrator to counter-act own-age bias. Our findings therefore strengthen support for the view that poorer lineup discriminability of older participants results from a decline in episodic memory with age (Colloff et al., 2017). This is in line with a body of literature showing that episodic memory is reduced in older adults (e.g. Hedden & Gabrieli, 2004; Nyberg et al., 2003; Nyberg & Tulving, 1996; Rӧnnlund et al., 2005).

#### Did describing the perpetrator affect discriminability?

Previous work on verbal overshadowing, with the exception of Wilson et al. (2018), has investigated identification in target-present lineups only and identified verbal overshadowing as a decrease in correct identifications after providing a description of the perpetrator. We, first, compare our findings directly with these studies by considering just our data from the target-present lineups and then, second, discuss our findings from our ROC analyses that enabled us to explore the effect of describing the perpetrator on discriminability.

Both our young and older groups made significantly fewer correct identifications on the target-present lineup in the experimental condition, in which they had to provide a description of the perpetrator prior to making their lineup identification decision, compared with the control condition, in which a description was not given. This verbal overshadowing effect is consistent with previous studies that have reported verbal overshadowing in correct identification in young participants (e.g. Schooler & Engstler-Schooler, 1990; Meissner & Brigham, 2001; Algona et al., 2014; Wilson et al., 2018). In addition to replicating these previous findings, it also demonstrates that verbal overshadowing generalises beyond the original stimulus materials used by Schooler and Engstler-Schooler (1990) that have been used in many subsequent studies, including the large registered replication of Alogna et al. (2014) and the experiments described in Wilson et al. (2018). Furthermore, we demonstrate verbal overshadowing on correct identification for the first time in older adults. Previous studies failed to find verbal overshadowing in this age group (Kinlen et al., 2007; Memon & Bartlett, 2002), which may have been due to limited power associated with small sample sizes in this previous work. This limitation was overcome by the larger sample tested in our study.

To determine whether the effect of verbal overshadowing on correct identification occurred because describing the perpetrator increased the difficulty of distinguishing between innocent and guilty suspects (decreased discriminability) or because it made participants less willing to pick a face from the lineup (i.e. more conservative response criterion), we used ROC analyses that can distinguish between these possibilities (see Mickes et al., 2012; Wixted & Mickes, 2014; Wilson et al., 2018).

For both the young and older participants, discriminability was lower in the experimental condition than the control condition, but the difference was not significant. Thus, we found no evidence in our study that describing the perpetrator made it more difficult to distinguish between innocent and guilty suspects, i.e. it did not significantly decrease discriminability. Our findings do not, therefore, provide support for theories, such as retrieval-based interference (RBI) theory (Meissner et al., 2001), that explain verbal overshadowing in terms of change to the memory trace. Rather the findings from our study suggest that describing the perpetrator led to a change to a more conservative criterion. We found that describing the perpetrator significantly reduced both correct identifications in the target-present lineup and false alarms in the target-absent lineup.

A similar pattern of correct identifications and false alarms was reported by Wilson et al. (2018), but this was accompanied by a significant decrease in discriminability in Experiments 2 and 4. Considered together, our findings and those of Wilson et al. (2018) demonstrate that both a decrease in discriminability and a change in criterion can result in verbal overshadowing on correct identification. This highlights the importance for interpreting the verbal overshadowing effect of using measures that distinguish discriminability and criterion.

Our finding that describing the perpetrator affects the criterion but not discrimination has two explanations within the framework of signal detection theory. The first explanation is a change of response bias (see Clare & Lewandowsky, 2004). Describing the perpetrator may make participants less willing to choose a face from the lineup perhaps due to the subjective difficulty of generating an adequate description (Clare & Lewandowsky, 2004). The second explanation is that the distributions of internal activity due to targets and fillers have both shifted in the same direction by the same amount (Witt et al., 2015; Wixted & Stretch, 2000). These two explanations are behaviourally indistinguishable.

Given the differing findings of our study and those of Wilson et al. (2018) it will be important, through further research, to characterise when and why verbal overshadowing results from a change in discriminability and when and why it results from a change in criterion. At this stage, it is hard to make specific practical recommendations in relation to these aspects of the results, but our findings reiterate the fact that simply asking someone to give a verbal description of a perpetrator may affect their future lineup ID decisions – something that is important for policymakers to bear in mind.

### Predictors of suspect identification accuracy

#### Confidence

High confidence responses were found to be more accurate than lower confidence responses for both age groups and for both conditions suggesting that confidence was informative about accuracy for both young and older participants. For both young and older participants, identifications made with high confidence were highly accurate. This was the case for both the control and experimental conditions. Accuracy decreased in both age groups as confidence decreased.

Our findings are consistent with previous studies that have shown that the confidence of young adult witnesses (Brewer & Wells, 2006; Grabman et al., 2019; Juslin et al., 1996; Seale-Carlisle 2019a, 2019b; Semmler et al., 2018; Wilson et al., 2018) and older witnesses (Colloff et al., 2017) when they make an initial identification is informative about the accuracy of their choice. While the majority of these studies assessed identification under conditions most comparable to our control condition, Wilson et al. (2018) also demonstrated this relationship when their young participants made their identification after first describing the perpetrator. Our finding that confidence predicted accuracy of identification in both our experimental and control conditions for our young participants therefore replicated the findings of Wilson et al. using different stimuli and participants.

Our finding that confidence predicted accuracy of identification in our older group in the control condition is consistent with the findings of Colloff et al. (2017) and shows that this relationship generalises to different stimuli and a longer retention interval than was used in that study. As shown in Fig. 2A, C, like Colloff et al. (2017), we found that high confidence was associated with high accuracy for our older participants even though our ROC analysis showed that their overall memory performance was poor compared to the young adults and this pattern was found in both the control and the experimental condition. Our findings therefore provide further evidence that even though older adults may have poorer memory overall than young adults, when they indicate that they are very confident about their identification, they are likely to be correct. This has implications for interpretation of eyewitness identifications in courts of law as it suggests that initial identifications made with high confidence by older witnesses are likely to be accurate. Inspection of the point sizes in Fig. 2A and frequencies in Table 2, which show that older participants made fewer high confidence identifications than young participants, suggest that older adults also have insight into the limitations of their memory and adjust their confidence ratings accordingly.

#### Response time

In the control condition, faster responses were associated with more accurate decisions in both the young and older participants. This is shown very clearly in Fig. 3A which plots the RAC curves for the two age groups in the control condition. Young participants were reliably more accurate for responses that were made in less than 6 s than for all slower responses, while older participants were reliably more accurate for responses made in less than 6 s, and those that took between 6 and 12 s to make, than for the longest responses, i.e. those that took more than 18 s to make. These findings are consistent with other studies that have found that the speed with which young adult witnesses make their initial lineup identification decisions is informative about the accuracy of their responses, with faster responses being more accurate (e.g. Brewer et al., 2006; Dodson & Dobolyi, 2016; Dunning & Perretta, 2002; Sauerland & Sporer, 2009; Seale-Carlisle et al., 2019a, 2019b; Smith et al., 2001; Sporer, 1992, 1993; Weber et al., 2004). Our data extend these findings by showing that this is also the case for older adults.

Furthermore, for the older participants, this relationship was maintained in the experimental condition, showing that response time remained informative of accuracy after describing the perpetrator for this age group. This was not, however, the case for the young group, whose mean performance remained high even for the longest response times, although the error bars were large. We cannot currently explain why response time only informed accuracy in this condition for the older participants. Determining whether this reflects a genuine age difference or an idiosyncrasy of our particular sample awaits further research. The smaller number of correct and incorrect identifications made in this condition may have been a contributory factor.

Like confidence, the speed with which participants made their identification was informative of accuracy for both the young and older adults. This demonstrates the importance of recording and using information about the speed with which witnesses make their initial identification in criminal cases. Considering identification speed, as well as the witness’s confidence in their identification, will provide additional informative measures to help judges and jurors determine the accuracy of the witness’s identification. A caveat though is that our data suggested that describing the perpetrator may have a disruptive effect on the relationship between response time and accuracy, as no relationship was found in this condition for our young participants. While the reliability of this finding requires further research, it raises the possibility that response time may be less informative of accuracy after describing the perpetrator, and so has implications for procedures for collecting evidence in criminal cases.

#### Future work

To ensure sufficient power for our ROC analyses we used a single video in our study that was viewed by all participants. This was different to the videos used in previous verbal overshadowing studies, many of which have used the original stimuli of Schooler and Engstler-Schooler (2009), enabling us to demonstrate that verbal overshadowing on correct identification generalises beyond the materials used in this previous work. Future work, however, will be required to determine to what extent the specific findings from our study generalise to other stimulus materials, e.g. different crime scenarios, different perpetrators and lineup filler faces. In particular, it will be important to characterise when and why verbal overshadowing results from a change in discriminability and when it results from a change in criterion.

## Conclusions

We found that older adults were poorer at discriminating the perpetrator from innocent suspects 25 min after viewing a video of a mock crime when own-age effects were controlled suggesting that poorer episodic memory in older adults may impact lineup identification accuracy. However, our confidence and response time data showed that when older adults were highly confident of their identification, and when they made their identification quickly, they were very accurate. This suggests that identifications made by older adults should not be dismissed in criminal cases on the basis of their age as they will not always be inaccurate. Rather confidence and identification speed should be taken into consideration as this will be informative about the likely accuracy of identifications made by both young and older adults. As reported in previous studies, we found that young adults were poorer at correctly identifying the perpetrator in a lineup after providing a description of him (verbal overshadowing) and demonstrated for the first time this effect in older adults. For both age groups, this effect was not due to an increase in difficulty distinguishing between innocent and guilty suspects (decreased discriminability) but rather resulted from a change in criterion which could be explained by participants adopting a more conservative response bias after describing the perpetrator. This finding highlights the importance of using measures that distinguish between discriminability and change in criterion when interpreting lineup identification data.

## Data Availability

The datasets supporting the conclusions of this article are available at https://doi.org/10.17605/OSF.IO/7EA23
